# Inhibition of Epidermal Growth Factor Receptor and PI3K/Akt Signaling Suppresses Cell Proliferation and Survival through Regulation of Stat3 Activation in Human Cutaneous Squamous Cell Carcinoma

**DOI:** 10.1155/2011/874571

**Published:** 2010-12-08

**Authors:** Toshinori Bito, Nahoko Sumita, Masashi Ashida, Arief Budiyanto, Masato Ueda, Masamitsu Ichihashi, Yoshiki Tokura, Chikako Nishigori

**Affiliations:** ^1^Division of Dermatology, Department of Clinical Molecular Medicine, Faculty of Medicine, Kobe University Graduate School of Medicine, 7-5-2 Kusunoki-cho, Chuo-ku, Kobe 650-0017, Japan; ^2^Department of Dermatology, University of Occupational and Environmental Health, Kitakyushu 807-8555, Japan

## Abstract

Recent studies have emphasized the important role of Stat3 activation in a number of human tumors from the viewpoint of its oncogenic and antiapoptotic activity. In this study, we examined the role and related signaling molecules of Stat3 in the carcinogenesis of human cutaneous squamous cell carcinoma (SCC). In 35 human cutaneous SCC samples, 86% showed overexpression of phosphorylated (p)-Stat3, and most of those simultaneously overexpressed p-EGFR or p-Akt. Constitutive activation of EGFR and Stat3 was observed in three SCC cell lines and four of five SCC tissues. AG1478, an inhibitor of the EGFR, downregulated Stat3 activation in HSC-1 human SCC cells. AG1478 inhibited cell proliferation and induced apoptosis of HSC-1 cells but did not inhibit the growth of normal human epidermal keratinocytes that did not show Stat3 activation. Furthermore, a PI3K inhibitor also suppressed Stat3 activation in HSC-1 cells to some degree. Combined treatment with the PI3K inhibitor and AG1478 strongly suppressed Stat3 activity and dramatically induced apoptosis of HSC-1 cells. These data suggest that Stat3 activation through EGFR and/or PI3K/Akt activation plays a critical role in the proliferation and survival of human cutaneous SCC.

## 1. Introduction

Stat3 activation has important implications in the cell proliferation of cutaneous squamous cell carcinoma (SCC). Knockdown of Stat3 activation completely prevents cell proliferation and growth in cutaneous SCC but is not sufficient to induce cell death [1]. Signaling cross talk may be responsible for the survival mechanism of SCC [2, 3]. 

 The epidermal growth factor receptor (EGFR) is a member of the ErbB family, which consists of four members: EGFR, ErbB2, ErbB3, and ErbB4. In particular, EGFR and ErbB2 have been implicated as therapeutic targets in various human cancers [4]. Receptor activation of the EGFR leads to the recruitment and phosphorylation of several downstream intracellular substrates, leading in turn to mitogenic signaling and other tumor-promoting cellular activities [5]. Over-expression of the EGFR in epithelial tumors, including head and neck, lung, breast, colon, and other solid malignant tumors, has frequently been correlated with their poor prognosis [6–9]. In breast cancers and head and neck SCC (HNSCC), a strong correlation between Stat3 and EGFR expression has been observed, and it has been suggested that the JAK/STAT pathway is one of the important downstream routes for EGFR signaling [10]. The recruitment sites of Stat3 within the EGFR have been identified and characterized, indicating the direct association of these two molecules [11]. In addition, the existence of EGFR independent signaling for Stat3 activation has been reported, enhancing the importance of Stat3 activation in tumor growth [11]. These data lead to the idea that Stat3 activation is very important for malignant proliferation of the epithelial tumors, and that EGFR activation correlates closely with Stat3 activation and is one of the key regulators. 

 Phosphotidylinositol-3 kinase (PI3K) is one of the downstream signaling molecules of the EGFR and plays a role in the proliferation or invasion of SCC [12, 13]. PI3K is an important factor in the development and progression of HNSCC [14]. There are multiple pathways that convert PI3K to the active form. Constitutive activation of PI3K by mutation and inactivation of its encoding gene is observed in a significant number of cancers [15]. Antagonists of EGFR, PI3K, and MEK have inhibitory effects on the growth of HNSCC [16]. However, there is little information on the role of PI3K/Akt and Stat3 pathways in mediating cell proliferation and the correlation between Stat3 and PI3K/Akt signaling in cutaneous SCC. 

 In this study, we aimed to investigate the activity of Stat3-related activators such as EGFR and PI3K/Akt in the proliferation of a cutaneous malignant tumor, SCC, and evaluate the therapeutic value of inhibition of the signaling pathways.

## 2. Materials and Methods

An EGFR inhibitor (AG1478) and a phosphotidylinositol-3 kinase (PI3K) inhibitor (wortmannin) were purchased from Calbiochem (San Diego, CA). Other reagents were from Sigma (St. Louis, MO). 

### 2.1. Patients and Tissue Sections

Samples of SCC, including three metastasis cases and adjacent skin, were obtained from 32 patients, 16 males and 16 females, with an average age of 74 years (range: 41–101 years). All subjects provided written informed consent prior to enrollment in the study. Surgically removed tissue samples were fixed in 10% neutral buffered formaldehyde and paraffin embedded for histopathology or immediately frozen in liquid nitrogen for nuclei extracts.

### 2.2. Cells and Cell Culture

Three human cutaneous SCC cells lines, HSC-1, -3, and -4, were kindly provided by Dr. Katagaka of the Yamagata University School of Medicine [17]. The immortalized human keratinocyte cell line, HaCaT, was kindly provided by Dr. Fusenig [18]. These cells were grown in Dulbecco's modified Eagle's medium (Life Technologies, Gaithersburg, MD) supplemented with 10% fetal bovine serum, 100 U/mL penicillin, and 100 *μ*g/mL streptomycin. Primary human epidermal keratinocytes (HEKs) were purchased from KURABO (Osaka, Japan). HEKs were grown in keratinocyte serum-free medium (SFM) supplemented with human keratinocyte growth supplement and PSA (penicillin, streptomycin, and amphotericin B) solution (Gibco, Grand Island, NY). Cells were maintained in a standard culture incubator with humidified air containing 5% CO_2_ at 37°C.

### 2.3. Immunohistochemistry

Skin specimens of 5 *μ*m paraffin-embedded sections were stained immunohistochemically using a streptavidin-biotin-peroxidase complex procedure provided by the DAKO CSA System, HRP (DAKO, Carpinteria, CA), according to the manufacturer's protocol. In brief, the sections were incubated at room temperature for 30 minutes with an anti-phospho-EGFR monoclonal antibody (mAb) (Tyr1173) (53A5), an anti-phospho-Akt (Ser473) (D9E) mAb, or a phospho-Stat3 (Tyr705) antibody (Cell Signaling Technology, Beverly, MA). After three washes with PBS, the samples were incubated with biotinylated antirabbit IgG and the avidin-biotin complex at room temperature for 15 minutes. Staining was completed by a 5-minute incubation with 3, 3'-diaminobenzidine tetrahydrochloride.

### 2.4. Electrophoretic Mobility Shift Assay (EMSA)

Nuclear extracts from surgical tissues were purified following the method described by Corsini et al. [19], with slight modifications. The tissues were frozen immediately in liquid nitrogen, and then 8 *μ*m slices from the tissue were cut with a cryostat. The sections were washed with PBS and were homogenized in 1 mL of a hypotonic lysis buffer (10 mM HEPES, pH 7.8, 10 mM KCl, 2 mM MgCl_2_, 1 mM dithiothreitol, 0.1 mM ethylenediaminetetraacetic acid, and 0.1 mM phenylmethylsulfonyl fluoride) with a tissue homogenizer for 20 seconds. Homogenates were kept on ice for 15 minutes, then 125 *μ*L Nonidet P-40 solution was added and mixed for 15 seconds, and the mixture was then centrifuged for 30 seconds at 12000 rpm. Nuclear extracts from the pellets or cells were prepared and EMSAs were performed essentially as described earlier [20]. Binding reaction mixtures (20 *μ*L) containing 5 *μ*g nuclear extract protein, 2 *μ*g poly (dI-dC) (Amersham Pharmacia Biotech, Piscataway, NJ, Sweden), ^32^P-labeled probe (Stat3), 50 mM NaCl, 2 mM MgCl_2_, 0.2 mM Na_2_EDTA, 1 mM DTT, 10% (v/v) glycerol, and 4 mM Tris-HCl (pH 7.9) were incubated for 30 minutes at room temperature. Proteins were separated by electrophoresis in native 6% polyacrylamide gels using a Tris-borate-EDTA running buffer (12.5 mM Tris-borate containing 0.25 mM Na_2_EDTA, pH 8.0), followed by autoradiography. The Stat3 probe (Santa Cruz Biotechnology, Santa Cruz, CA) 5'…GAT CCT TCT GGG AAT TCC TAG ATC…3'; 3'…CTA GGA AGA CCC TTA AGG ATC TAG…5' was labeled with [*γ*-^32^P] dATP (Du Pont NEN, Boston, MA) using T4 polynucleotide kinase (Boehringer MannheimRoche, Mannheim, Germany).

### 2.5. Immunoprecipitation and Immunoblot Analysis

The cells were washed with ice-cold D-PBS containing 200 *μ*M sodium orthovanadate (Na_3_VO_4_), frozen immediately in liquid nitrogen, and then lysed in lysis buffer (25 mM Tris-HCl, pH 7.6, 200 mM boric acid, 150 mM NaCl, 50 mM NaF, 5 mM Na_2_EDTA, 1% Triton X-100, 10 mM sodium pyrophosphate, 2 mM EGTA, 20 mM p-nitrophenyl phosphate, 1% BSA, 20 *μ*M Na_3_VO_4_, and 2 mM DTT) containing protease inhibitors (2 *μ*g/mL each of aprotinin, leupeptin, pepstain, antipain, and 100 *μ*g/mL PMSF). The lysates were centrifuged at 10,000 × g at 4°C for 15 minutes, and the resulting supernatants were subjected to immunoprecipitation or immunoblot analysis. Protein concentrations in the supernatants were quantitated using the Coomassie plus protein assay reagent (Bio-Rad Loboratories, Tokyo, Japan). Samples (20 *μ*g protein each) for immunoblotting were separated on 8% SDS-polyacrylamide gel electrophoresis gels for Akt and Stat3 and were blotted onto Hybond-ECL nitrocellulose membranes (Amersham Pharmacia Biotech, Piscataway, NJ, USA), followed by blocking with 5% skim milk in Tris-buffered saline for 2 hours at room temperature, and were then probed with a primary antibody to phospho-Akt (Ser473), phospho-Stat3 (Tyr705 or Ser727) antibody (Cell Signaling Technology), or a horseradish peroxidase (HRP)-conjugated anti-phosphotyrosine antibody, PY20 (Santa Cruz Biotechnology, Santa Cruz, CA), overnight at 4°C. Bound antibodies were detected using the ECL Western blotting detection reagent (Amersham Pharmacia Biotech). After film exposure, the membranes were washed 4 times for 5 minutes each in PBS-Tween 20 and were then incubated for 30 minutes at 50°C in stripping buffer (62.5 mM Tris HCl pH 6.8, 2% SDS, and 100 mM 2-mercaptoethanol). The membranes were blocked with 5% skim milk again, and then reprobed with an antibody to Akt antibody or Stat3 antibody (Cell Signaling Technology) or reblotted with human actin (Santa Cruz) as a loading control overnight at 4°C. Supernatants for immunoprecipitation were incubated at 4°C with the anti-EGFR (1005) antibody (Santa Cruz) bound to G-Sepharose beads (2 *μ*g Ab per 20 *μ*L beads; Amersham Pharmacia Biotech) for 4 hours, after which the beads were washed twice with 1 mL WG buffer (50 mM HEPES-NaOH, pH 7.6, 150 mM NaCl, 0.1% Triton X-100) and resuspended in SDS sample buffer. Quantification of protein expression was done using ImageJ analysis software (version 1.44b) (Bethesda, MD, USA).

### 2.6. Cell Proliferation Assay

Cell growth was determined using the MTS cell proliferation assay (Promega, Madison, WI). Briefly, HSC-1 cells or HEK were cultured in 96 well plates. Twenty-four hours after seeding, the cells were treated with AG1478 (0.25 or 2.5 *μ*M) for the indicated time and the medium and substrates were exchanged every 24 hours, after which the MTS reagent was added and allowed to react for 2 hours. Absorbance at 490 nm was measured using a microplate reader (Emax; Molecular Devices, Sunnyvale, CA). Cell growth is expressed as a percentage of that in nontreated cells at each indicated time point. DMSO was used as a control for AG1478 treatment. To assess the effect of serum on cell growth, the cells were seeded at a density of 1 × 10^4^ cells/well in the medium with serum in 96 well plates. The medium was exchanged every 24 hours for medium with or without serum. Cell growth was evaluated at 0 hour, 36 hours, and 48 hours after the first exchange of medium. The results are expressed as a percent ratio of serum-free to serum conditions for HaCaT and HSC-1 cells.

### 2.7. Detection of Cell Death

Apoptosis of cells was analyzed by flow cytometry. Early in apoptosis, membrane phospholipid phosphatidylserine (PS) is translocated to the outer leaflet of the plasma membrane. Annexin V is a 35-36 kD Ca^2+^ dependent, phospholipid-binding protein that has a high affinity for PS. Staining with Annexin V-FITC (BD Pharmingen, San Diego, CA) in conjunction with the vital dye propidium iodide (PI) (Sigma) was performed to identify early apoptotic cells. HSC-1 cells were treated with control solvent or with AG1478 (0.25 or 2.5 *μ*M) for 12 hours. Cells were scraped, then spun down, and washed twice with 5 mL cold PBS. The cells were resuspended in binding buffer (0.01 M HEPES, pH 7.4; 0.14 M NaCl; 2.5 mM CaCl_2_) at a concentration of 1 × 10^6^ cells/mL and then stained according to the manufacturer's protocol. Cells were analyzed using a FACS caliber flow cytometer (BD, San Jose, CA). Apoptotic cells exclude all those dyes which are used in cell viability assays, such as PI, while necrotic cells do not. In cells with a damaged cell membrane, PI induces a red fluorescence of the DNA, while it is excluded from cells with a preserved plasma membrane [21]. The lower left quadrant (LL) of the cytograms shows the viable cells, which exclude PI and are negative for Annexin V-FITC binding. The upper right quadrant (UR) represents nonviable, necrotic cells, positive for Annexin V-FITC binding and PI uptake. The lower right quadrant (LR) represents apoptotic cells, Annexin V-FITC is positive but PI is negative.

### 2.8. Statistical Analysis

Data are expressed as mean ± standard deviation of at least three separate experiments. Statistical analyses were performed using Prism 5 software (GraphPad Software, San Diego, CA). Unpaired experimental groups were compared with the Student's *t*-test. Value of *P* < .05 was considered to be statistically significant.

## 3. Results

### 3.1. EGFR, Akt, and Stat3 Are Frequently Overexpressed in Human Cutaneous SCC Tissues

**Figure 1:**
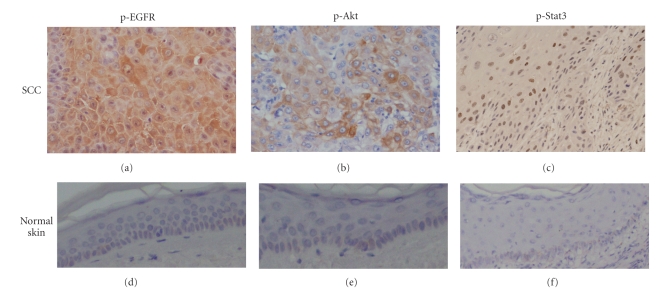
Over-expression of p-EGFR, p-Akt, and p-Stat3 in human cutaneous SCC tissues. Paraffin-embedded serial sections of SCC from the same patient were examined immunohistochemically for p-EGFR, p-Akt, and p-Stat3 (tyr705) expression. Characteristic immunostaining of p-EGFR, p-Akt and p-Stat3; original magnification: ×200. P-EGFR (a) and p-Akt (b) protein were overexpressed mainly in the cytoplasm and partially in the nucleus. P-Stat3 (tyr705) protein was detected in the nuclei (c). None of p-EGFR p-Akt or p-Stat3 protein were seen in normal skin ((d), (e), (f)); original magnification: ×100.

Thirty-five paraffin-embedded sections of SCC including 2 metastatic lesions were examined immunohistochemically for the expression of phosphorylated (p)-EGFR, p-Akt, and p-Stat3, and the cases in which they were expressed in more than 20% of the cells were defined as positive (Figure 1, Table 1).

Twenty-five of the 35 SCCs showed positive expression of p-EGFR in tumor cells. The reactivity of p-EGFR was seen mainly in the cytoplasm and partially in the nucleus (Figure 1(a)). P-Akt was expressed in the cytoplasm and nucleus of atypical cells (Figure 1(b)). Stronger p-Akt expression was seen in atypical cells surrounding the invasive lesions into the dermis. We evaluated Stat3 activation by the expression of both phospho-Tyr705 and phospho-Ser727. Most of the cases showed phosphorylation on both sites, mainly in the nuclei, and partially in the cytoplasm, though the expression at each site differed in degree. Overall, expression of Tyr705 was considered to be positive for p-Stat3 (Figure 1(c)). Thirty of the 35 SCCs showed a positive reaction for p-Stat3. Actinic keratosis, a premalignant lesion of SCC, also over-expressed p-Stat3 if the adjacent SCC was also positive for p-Stat3. None of p-EGFR, p-Akt, or p-Stat3 were seen in tissue adjacent to the tumor or normal skin (Figures 1(d), 1(e), and 1(f)). Twenty-seven of 30 p-Stat3 positive cases over-expressed both or either of p-EGFR and p-Akt, and three of the 30 cases showed negative for both p-EGFR and p-Akt expression. Eight of 35 SCCs showed over-expression of p-EGFR and p-Stat3 without p-Akt over-expression. In contrast, only two of the 35 cases showed over-expression of p-Akt and p-Stat3 without p-EGFR over-expression. Two of the 3 metastatic lesions over-expressed p-Stat3 as well as p-EGFR and p-Akt (Table 1). 

**Figure 2:**
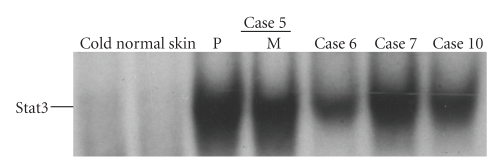
Analysis of Stat3 activation in human cutaneous SCC tissues. Nuclear extracts were generated from surgical tissues. In case 5, normal skin, primary lesion (P), and metastatic lesion (M) of SCC from the same patient were analyzed for Stat3 activation using EMSA with a ^32^P-labeled DNA probe to detect binding activity of Stat3. Specificity of the DNA-binding complex was evaluated by competition with excess unlabeled Stat3 oligonucleotide (cold). Stat3 activation of the primary lesions of the other 3 cases was also analyzed. Three of the 4 primary lesions and a metastatic SCC showed the strong DNA binding activity of Stat3 while little activation was seen in normal skin samples examined and in one primary SCC lesion (case 6). Data of normal skin are representative of 4 normal skin samples.

Next, to evaluate the functional activity of p-Stat3 protein expression *in vivo*, the Stat3 DNA binding activity of SCC tumors and normal skin was investigated. Four of 5 human SCC tissues showed strong Stat3 DNA binding activity while no DNA binding activity was seen in normal skin samples (Figure 2). The results of Stat3 DNA binding activity were consistent with those of the immunohistochemical study; that is, all of the 4 tissues with positive DNA binding activities were positively stained for p-Stat3 by immunohistochemistry and one case without DNA binding activity was negative for protein expression (Table 1).

### 3.2. EGFR and Stat3 Are Activated in Human SCC Cell Lines but Less in Human Epidermal Primary Keratinocytes or in HaCaT Cells

**Figure 3:**
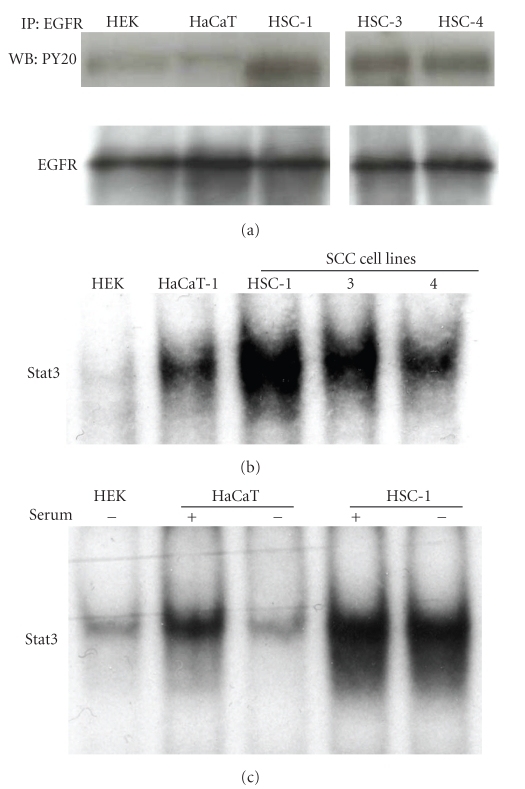
Analysis of EGFR and Stat3 activation in human epidermal primary keratinocytes (HEK), HaCaT cells, and human SCC cells. Cells were incubated in serum-free medium for 24 hours, and then cell lysates were extracted from HEK, HaCaT cells, HSC-1, -3, or -4 cells. Total 100 *μ*g of protein in each lysate were subjected to immunoprecipitation (IP) with the anti-EGFR antibody. The immunoprecipitates were separated by SDS-PAGE and subjected to immunoblot analysis with an anti-phosphotyrosine antibody, PY20. The same membrane was then reprobed with the anti-EGFR antibody. Data are representative of three independent experiments (a). Nuclear extracts from HEK, HaCaT cells, HSC-1, -3, or -4 cells under the regular condition were analyzed using EMSA with a ^32^P-labeled DNA probe to detect DNA binding activity of Stat3 (b). HSC-1 or HaCaT cells were seeded in medium with 10% fetal calf serum, and the cells were cultured for 24 hours, after which they were cultured in medium with or without 10% fetal calf serum for 24 hours and Stat3 activation was analyzed using EMSA (c).

The status of EGFR and Stat3 phosphorylation was investigated in HEK, HaCaT, and human SCC cell lines (HSC-1, -3, and -4). Cells were cultured in serum-free medium for 24 hours before harvest, and then lysates were utilized for immunoprecipitation or immunoblotting. HSC-1, -3, and -4 cells showed strong expression of p-EGFR while HEK, and HaCaT cells showed little p-EGFR expression (Figure 3(a)). Although the medium for HEK contains EGF (1~2 ng/mL), no detectable EGFR phosphorylation was observed in HEK (Figure 3(a)). The DNA binding activity of Stat3 was analyzed using EMSA. Constitutive activation of Stat3 was observed in HaCaT, HSC-1, -3, and -4 cells under conventional culture conditions (Figure 3(b)). HaCaT cells showed little Stat3 DNA binding activity when they were cultured in serum-free medium while the addition of fetal calf serum activated Stat3 within 24 hours (Figure 3(c)). In contrast, cultivation of HSC-1 cells in serum-free medium reduced little amount of the Stat3 DNA binding activity (Figure 3(c)). The basal activity of Stat3 in HEK was very low (Figures 3(b) and 3(c)).

### 3.3. Inhibitor of EGFR Downregulates the Constitutive Activation of Stat3 in HSC-1 Cells, and a Combination of Inhibitors of EGFR and PI3K/Akt Abrogates Stat3 Activation

**Figure 4:**
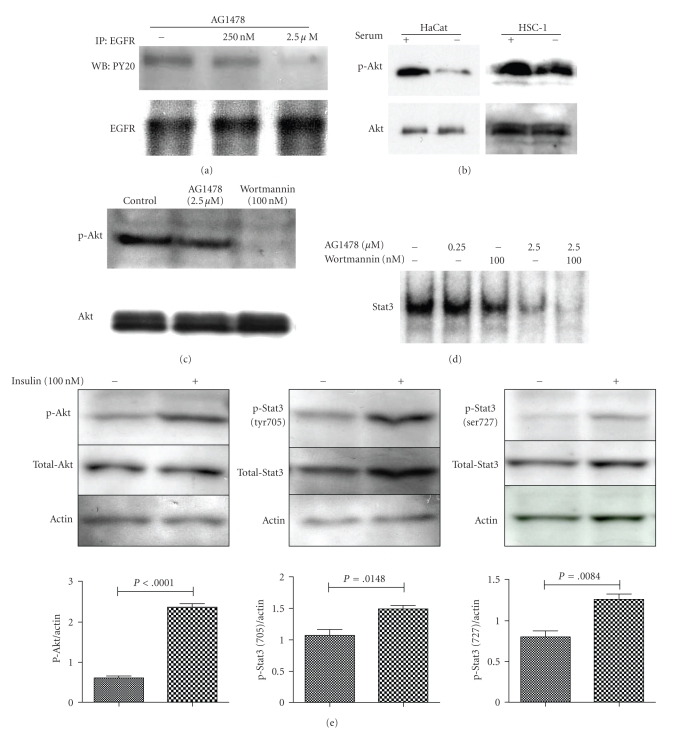
*Effect of wortmannin, AG1478, and insulin on activation of Stat3 and Akt in HSC-1 cells. *HSC-1 cells were incubated in serum free medium for 24 h, and were then treated with AG1478 at the indicated concentration for 1 h. The cell lysates were subjected to IP with the anti-EGFR antibody. The same membrane was then reprobed with the anti-EGFR antibody (a). The cells were incubated with or without serum for 24 h, and then each lysate was immunoblotted with anti-p-Akt antibody, and then reprobed with the anti-Akt antibody (b). HSC-1 cells were incubated in serum free medium for 24 h, and were then treated with AG1478 or wortmannin for 1 h. The cell lysates were immunoblotted with the anti-p-Akt antibody, and then reprobed with the anti-Akt antibody (c). HSC-1 cells were incubated in serum free medium for 24 h, and were then treated with AG1478 and/or wortmannin at the indicated concentration for 1 h. DNA binding activity of Stat3 was analyzed using EMSA (d). HSC-1 cells were incubated without serum for 24 h, and were then treated with or without insulin for 15 min. The cell lysates were immunoblotted with each of the anti-p-Akt or p-Stat3 antibody and then reblotted with human actin as a loading control. The same membrane was reprobed with the anti-Stat3 or Akt antibody (e). The bar graphs are expressed as the relative ratio of p-Akt or Stat3 protein expression to actin protein expression by densitometry. Data are mean ± SD of three independent experiments. *P* < .05 are statistically significant when compared with the non-treated control (e).

The correlation between Stat3 activation and EGFR activation was examined using the EGFR-specific inhibitor tyrphostin AG1478. Expression of p-EGFR was reduced by treatment with AG1478 (Figure 4(a)). Constitutive activation of Stat3 in HSC-1 cells was also downregulated by AG1478 in a dose-dependent manner (Figure 4(d)). However, the DNA binding activity of Stat3 was still observed in HSC-1 cells after treatment with 2.5 *μ*M of AG1478, indicating that alternative pathways are involved in Stat3 activation in HSC-1 cells. To explore the distinct signal that activates Stat3 in SCC, Akt activation was investigated by immunoblot analysis. Low expression of p-Akt and p-Stat3 protein was observed in HEK and HaCaT cells in serum-free conditions (data not shown). Over-expression of p-Akt protein as well as p-Stat3 protein was observed in SCC cell lines. P-Akt protein expression was slightly reduced after 24-hour serum starvation in HSC-1 cells, but the Akt activity was still maintained even under serum-free conditions. In contrast, the dramatically reduced level of p-Akt expression was observed in HaCaT cells in serum-free conditions (Figure 4(b)). We investigated the role of PI3K/Akt signaling on Stat3 activation in HSC-1 cells. The activation of Akt was abrogated by treatment with a PI3K inhibitor, wortmannin, but not by treatment with AG1478 (Figure 4(c)). The effect of treatment with wortmannin on Stat3 phosphorylation and DNA binding activity was examined by EMSA in HSC-1 cells. Each treatment, respectively, suppressed the activity of Stat3 to some degree, and the combination of AG1478 and wortmannin showed an additive effect on the suppression of Stat3 DNA binding activity (Figure 4(d)). Next, the link between Stat3 activation and Akt activation was examined by immunoblotting with anti-phospho-antibody to Stat3 or Akt. After preincubation in the absence of serum for 24 hours, HSC-1 cells were incubated in the presence or absence of 100 nM insulin for 15 minutes and then lysed. Immunoblotting with the anti-phospho-antibody of Akt (ser 473), Stat3 (tyr705), or Stat3 (ser 727) and the total antibody of Akt or Stat3 showed synchronized upregulation of both proteins, and a stimulator of Akt activation, insulin, resulted in significant increase on phosphorylation of both Tyr705 and Ser727 (Figure 4(e)). P-Akt appeared to be a candidate as an activator of Stat3 in HSC-1 cells.

### 3.4. An EGFR Inhibitor Suppresses the Growth of HSC-1 Cells but Not That of HEK

**Figure 5:**
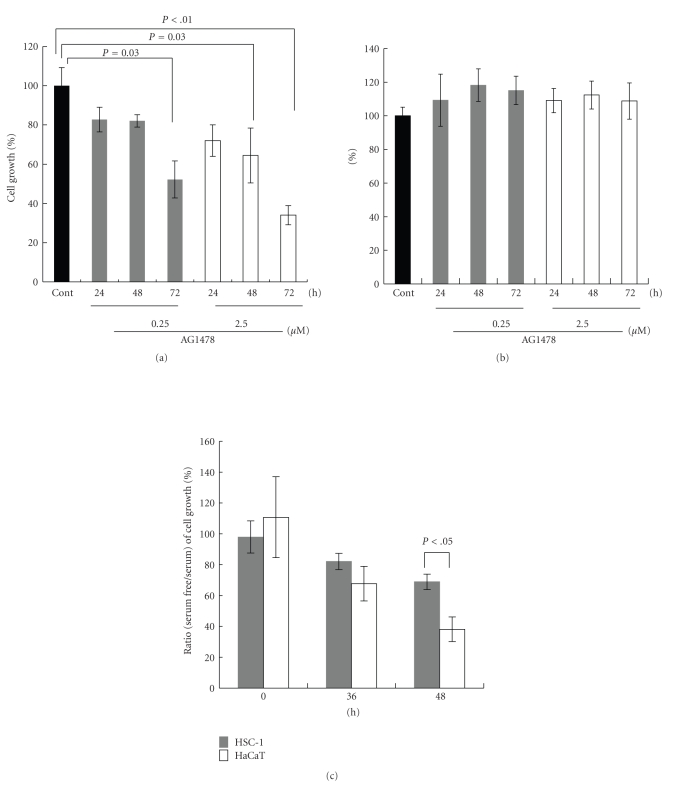
Effect of AG1478 on proliferation of HSC-1 cells and HEKs. After HSC-1 cells or HEKs were incubated with AG1478 (0.25 or 2.5 *μ*M) for indicated periods, cell proliferation was analyzed using the MTS assay ((a), (b)). Cell growth of HSC-1 cells (a) or HEKs (b) was expressed as a percentage of that in untreated cells at each indicated time point up to 72 hours. HSC-1 or HaCaT cells were seeded in medium with 10% fetal calf serum and were cultured for 24 hours. The medium was then changed to serum-free medium or medium with 10% fetal calf serum. The growth of HSC-1 and HaCaT cells was monitored using the MTS assay at 0 hour, 36 hours, and 48 hours after the change of medium and was expressed as the percent ratio (serum-free/serum) (c). Data are mean ± SD of at least three experiments. *P* < .05 when compared with each group.

The effects of AG1478 on the growth of HSC-1 cells and HEK were evaluated using the MTS assay. Cell growth is expressed as a percentage of that in untreated cells at each indicated time point. Treatment with AG1478 significantly suppressed the growth of HSC-1 cells (*P* < .01 or  .05) (Figure 5(a)). After 72 hours of AG1478 treatment at a concentration of 2.5 *μ*M, the cell density was suppressed significantly to 38% of untreated HSC-1 cells (*P* < .01) (Figure 5(a)). Alternatively, the same treatment did not affect the growth of HEK for at least 72 hours under culture conditions containing 1 or 2 ng/mL EGF (Figure 5(b)). HaCaT cells grew slowly in the serum-free medium. To examine the effect of serum in the culture medium on the growth of HSC-1 or HaCaT cells, they were seeded in medium with 10% fetal calf serum, after which the medium was exchanged for serum-free medium or medium with 10% fetal calf serum every 24 hours. The growth of HSC-1 and HaCaT cells was monitored using the MTS assay and was expressed as the percent ratio (serum-free/serum) (Figure 5(c)). The percent ratio (serum-free/serum) of cell growth at 48 hours was 38% and 71% in HaCaT cells and HSC-1 cells, respectively. The proliferation of HaCaT cells under serum-free conditions was significantly decreased at 48 hours (*P* < .05) (Figure 5(c)). These values were significantly different, indicating that the proliferation of HaCaT cells is stimulated more by serum.

### 3.5. An EGFR Inhibitor and a PI3K Inhibitor Induce Apoptosis in HSC-1, 3, and 4 Cells

**Figure 6:**
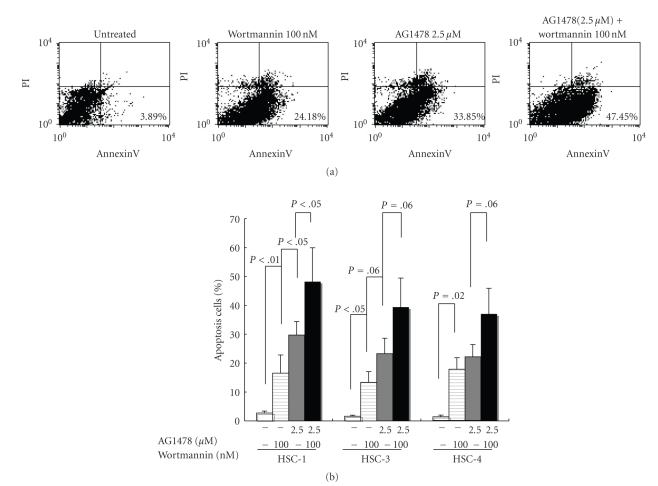
Analysis of apoptosis induced by AG1478 and wortmannin in HSC-1 cells. HSC-1 cells were incubated with AG1478 and/or wortmannin at the indicated concentration for 24 hours. Cells were incubated with Annexin V-FITC in a buffer containing propidium iodide (PI) and analyzed by flow cytometry (a). Data are representative of at least four independent experiments. Annexin V-FITC positive and PI negative cells are apoptotic (lower right quadrant) (a). Annexin V-FITC positive and PI positive cells are necrosis (upper right quadrant) (a). HSC-1, 3, and 4 cells were incubated with AG1478 (2.5 *μ*M) and/or wortmannin (100 nM) for 24 hours. Cells were incubated with Annexin V-FITC in a buffer containing propidium iodide (PI) and analyzed by flow cytometry. Results are means ± SD of at least three independent experiments (b). Statistical significance of the difference between each group was determined using Student's *t*-test and analysis of variance.

To elucidate the role of EGFR activation on apoptosis, double staining for Annexin V-FITC binding and for cellular DNA using PI was performed on HSC-1, 3, and 4 cells. In the control, 0.54% of the cells were apoptotic. Apoptotic cell populations were increased by treatment with AG1478 in a dose-dependent manner. AG1478 at doses of 0.025, 0.25, or 2.5 *μ*M increased the apoptotic cell population (data not shown). No induction of apoptosis by AG1478 was observed in HEK. The apoptosis was also induced in HSC-1 cells by treatment with a PI3K inhibitor, wortmannin (*P* < .01, when compared with untreated) (Figure 6(a)). The percentage of apoptotic cells induced by wortmannin at 100 nM was significantly (*P* < .05) lower than that by AG1478 at 2.5 *μ*M in HSC-1 cell lines (Figure 6(b)). The greatest induction of apoptosis (48.2%) (*P* < .05, when compared with AG1478) among the treatments tested in this study was obtained by the combined treatment of AG1478 at 2.5 *μ*M and wortmannin at 100 nM in HSC-1 cells (Figure 6(b)). PI staining showed that cell viability (UR) was not affected by these treatments (Figure 6(a)). Although a similar trend was observed in HSC-3 and 4 cells, the difference between apoptosis rates induced by each treatment in HSC-3 and 4 cells was not as big as in HSC-1 cells (Figure 6(b)).

## 4. Discussion

 This study demonstrated the crucial role of Stat3 in the proliferation of cutaneous SCC and the important contribution of the dual signaling of EGFR and PI3K/Akt on the activation of Stat3. *In vivo* analysis using immunohistochemistry and EMSA reveals that Stat3 activation is frequently observed in association with EGFR and/or Akt activation in human cutaneous SCC. Nearly 86% of human cutaneous SCCs expressed p-Stat3 as shown in Table 1. No preferential regions for p-Stat3 expression were found in this study. In accordance with *in vivo* results, we easily found three human cutaneous SCC cell lines, which showed constitutive Stat3 activation. Although Stat3 activation was not shown in all SCC cell lines, Stat3 appears to be activated in most cases of cutaneous SCCs.

 In agreement with studies emphasizing the critical role of EGFR on Stat3 activation in the carcinogenesis of mouse or human HNSCC [22, 23], we also demonstrated the importance of EGFR activation in Stat3 activation in human cutaneous SCC. It has been suggested that the molecular mechanisms for EGFR activation are autocrine regulation or gene amplification in other organ malignancies such as breast or brain tumors [24–26]. However, the autocrine mechanism may not be relevant in HSC-1 cells since the addition of a neutralizing antibody to EGF did not affect activation of the EGFR in the cells (data not shown). 

 A different reaction to serum regarding growth and Stat3 activation between HaCaT cells and HSC-1 cells was observed in our study. The Stat3 activity of HaCaT cells was markedly stimulated by serum, while that of HSC-1 cells was slightly stimulated (Figure 3(c)). These results indicate a close correlation between cell growth and Stat3 activation. In HaCaT cells, Stat3 activity was low in serum-free conditions and the addition of serum induced Stat3 activity. HSC-1 cells also showed a small decrease in Stat3 activity under serum-free conditions and accordingly there was a small additional activation following the addition of serum. In parallel to Stat3 activity, the addition of serum to cells cultured in serum-free conditions stimulated the proliferation of HaCaT cells to a greater extent than HSC-1 cells. A recent study by Quadros et al. [27] has shown that EGFR-dependent Stat3 activation is observed only in SCC cell lines but not in HaCaT cells, because EGF did not activate Stat3 in HaCaT cells. Since we did not add EGF but only serum and found Stat3 activation in HaCaT cells, other ligands or growth factors might have activated Stat3 in HaCaT cells. Regarding the Stat3 activation in normal keratinocytes, Quadros et al. [27] showed no activation by EGF in human keratinocytes whereas Chan et al. [22] showed its activation by EGF in mouse keratinocytes, indicating a species difference. Stat3 activation may be regulated in various ways according to species and cell type. 

 In our analysis of HSC-1 cells, the DNA binding activity of Stat3 was downregulated by AG1478 in a dose-dependent manner. However, in spite of the nearly complete reduction of EGFR expression following AG1478 treatment, Stat3 activation was still observed in HSC-1 cells. This suggests the presence of an EGFR-independent activation of Stat3 in HSC-1 cells. It was shown that gp130/JAK and SRC-like kinase contribute slightly to the EGFR-independent pathway of Stat3 activation in SCC cell lines [27]. Alternatively, the PI3K, catalytic, alpha gene is somatically mutated in several kinds of human cancer [28]. The response of Stat3 and Akt activation to serum stimulus between HaCaT and HSC-1 cells provided a clue. Akt was strongly activated by serum in HaCaT cells. In contrast, Akt activation was observed in HSC-1 cells under serum-free condition, suggesting persistent activation of PI3K/Akt signaling in cancer cells. Maximum suppression was achieved by a combination of AG1478 and wortmannin (Figure 4(d)), indicating that EGFR and PI3K/Akt signaling directly or indirectly activate Stat3 through independent pathways in HSC-1 cells. 

 Combined treatment of wortmannin and AG1478 showed an additive effect on the induction of apoptosis as well as on the suppression of Stat3 activity in all SCC cell lines (Figure 6). The induction of apoptosis in human SCC cells also appeared to be inversely proportional to Stat3 activity (Figures 3(b) and 6(b)). However, we have demonstrated that Stat3 inhibition is not sufficient to induce apoptosis, but Stat3 activation is required for cell proliferation and tumorigenesis of SCC [1]. Shih et al. reported that Stat3 works as a strong antiapoptotic agent of TGF-*β*-induced apoptosis in cooperation with PI3K/Akt [29]. In addition, Corney et al. revealed that Akt regulates the sensitivity to TGF-*β* through interaction with Smad3 [30]. These recent data may support the proapoptotic effect of the combination of inhibitors used in this study.

 We conclude that the proliferation and survival of SCC cells is mostly maintained by Stat3 activation and that Stat3 activation is mediated through EGFR and PI3K/Akt activation in cutaneous SCC cell lines. This study could provide clues to elucidate the carcinogenesis of cutaneous SCC and lead to a novel treatment and therapeutic strategies.

## Figures and Tables

**Table 1:** Immunohistochemical analysis of human cutaneous SCC tissues.

Case	Age	Sex	Site	p-EGFR	p-Akt	p-Stat3	EMSA Stat3
1	64	M	Back, primary lesion	+	−	+	N.D.
1′			Lymph node, metastasis of case 1	+	−	+	N.D.
2	89	F	Face	+	+	+	N.D.
3	41	F	Foot	−	+	−	N.D.
4	65	M	Head	+	+	+	N.D.
5	61	F	Hand, primary lesion	+	+	+	+
5′			Lymph node, metastasis of case 5	+	+	+	+
6	58	M	Thigh	−	+	−	−
7	81	M	Foot finger	+	+	+	+
8	54	F	Lower leg	−	−	−	N.D.
9	41	F	Hand finger	+	−	+	N.D.
10	72	M	Forehead	+	+	+	+
11	81	F	Face	+	−	+	N.D.
12	72	F	Face	+	+	+	N.D.
13	75	M	Face	+	+	+	N.D.
14	90	F	Face	−	−	−	N.D.
15	62	M	Face	+	+	+	N.D.
16	64	M	Face	+	+	+	N.D.
17	80	F	Face	−	−	+	N.D.
18	96	F	Face	+	+	+	N.D.
19	88	M	Head	−	+	+	N.D.
20	81	M	Lower leg	+	+	+	N.D.
21	77	M	Head	+	+	+	N.D.
22	89	F	Head	+	−	+	N.D.
23	71	F	Face	−	+	+	N.D.
24	93	F	Vulva	+	+	+	N.D.
25	71	M	Foot back	+	+	+	N.D.
26	81	M	Head	+	−	+	N.D.
27	55	F	Sole	−	−	+	N.D.
28	101	M	Face	+	−	+	N.D.
29	87	F	Forearm	+	−	+	N.D.
29′			Lymph node, metastasis of case 29	+	+	+	N.D.
30	73	M	Thigh	−	−	+	N.D.
31	87	M	Scrotum	+	−	+	N.D.
32	74	F	Lip	−	−	−	N.D.

			Positive cases/Total	25/35	20/35	30/35	4/5

+: positive, −: negative, N.D.: not done.

## Abbreviations

EGFR: Epidermal growth factor receptor
PI3K: Phosphotidylinositol-3 kinase
SCC: Squamous cell carcinoma
STAT: Signal transducer and activator of transcription.
